# Clinical characteristics and prognosis of ALL in children with CDKN2A/B gene deletion

**DOI:** 10.3389/ebm.2025.10447

**Published:** 2025-02-13

**Authors:** Yiyu Wang, Peijing Wu, Xiaoyan Mao, Nanjing Jiang, Yu Huang, Li Zhang, Li Liu, Xin Tian

**Affiliations:** ^1^ Department of Hematology, Sichuan Provincial Woman’s and Children’s Hospital/The Affiliated Women’s and Children’s Hospital of Chengdu Medical College, Chengdu, China; ^2^ Department of Pediatrics, Anning First People’s Hospital, Kunming, China; ^3^ Department of Pediatrics, Children Hematological Oncology and Birth Defects Laboratory, Sichuan Clinical Research Center for Birth Defects, The Affiliated Hospital of Southwest Medical University, Luzhou, Sichuan, China; ^4^ Department of Pediatrics, Da Li University, Da Li, China; ^5^ Department of Pediatrics, Qujing Medical College, Qujing, China

**Keywords:** CDKN2A/B gene, acute lymphoblastic leukemia, children, survival analysis, clinical characteristics

## Abstract

This study aimed to explore the correlation between the deletion of the CDKN2A/B gene and the prognosis of pediatric acute lymphoblastic leukemia (ALL) patients. A total of 310 pediatric patients who were diagnosed with acute lymphoblastic leukemia at our hospital from January 2020 to September 2023 were included in this study. Among them, 78 patients with CDKN2A/B deletion were included in the final analysis. Additionally, 78 ALL patients without CDKN2A/B deletion, who were diagnosed during the same period, were randomly selected for comparison. A statistical analysis was conducted to compare the clinical characteristics and prognosis between the CDKN2A/B deletion group and the non-deletion group in ALL patients. The results showed that pediatric ALL patients with CDKN2A/B deletion had higher white blood cell counts and a greater proportion of immature cells in peripheral blood at diagnosis. The age at diagnosis was older in the deletion group, with a greater proportion in the >10-year-old group. CDKN2A/B deletion occurred more frequently in pediatric patients with T-ALL than in pediatric patients with B-ALL. Patients with CDKN2A/B deletion were more likely to have positive BCR-ABL1 expression combined with IKZF1 deletion. The overall survival (OS) rate was 89.7%, and the event-free survival (EFS) rate was 83.3% in the CDKN2A/B deletion group, which was lower than the OS rate of 97.4% and EFS rate of 93.6% in the non-deletion group. These results suggest that CDKN2A/B deletion may be one of the factors affecting poor prognosis. It provides a new perspective for clinical treatment, risk stratification, and prognostic assessment in pediatric ALL patients.

## Impact statement

Research indicates that the CDKN2A/B gene is correlated with the occurrence, development, and prognosis of some tumors. However, there is no consensus or definitive conclusion regarding the clinical characteristics, biological manifestations, and prognosis of pediatric ALL patients with CDKN2A/B deletion. Further analysis and discussion are needed based on a large number of clinical samples and precise experimental data to elucidate the significance of CDKN2A/B deletion in the prognosis of pediatric ALL patients. This study aimed to explore the association between CDKN2A/B deletion and prognosis in pediatric ALL patients, with the goal of providing a new perspective for clinical treatment, risk stratification and prognostic assessment in pediatric ALL patients.

## Introduction

Acute lymphoblastic leukemia (ALL) is the most common malignant tumor disease in children [1]. While the majority of children with ALL can achieve a cure with conventional chemotherapy, 20% of these children still experience leukemia relapse. Relapsed ALL remains a leading cause of cancer-related death in children [2, 3]. The current first-line treatment is chemotherapy stratified by risk factors. With the development of genetic sequencing technology, an increasing number of risk factors associated with pediatric ALL have been identified to guide the risk stratification and treatment of ALL. Next-generation sequencing (NGS) technology is capable of detecting deeper levels of single nucleotide variations (SNVs), small insertions and deletions, and copy number variations (CNVs) [4]. The application of NGS in pediatric leukemia is becoming increasingly widespread [5]. When applied to leukemia diagnosis, certain meaningful gene mutations related to treatment, disease progression, prognosis, and risk factors of relapse and refractory can be detected earlier and more comprehensively than traditional gene detection methods [6]. This, in turn, provides better and timelier strategies and precision treatment for pediatric ALL patients, with the expectation of improving the therapeutic outcomes and prognosis of children with ALL.

With the application of NGS detection technology, numerous studies are being conducted to explore the correlation between copy number variations and the prognosis of pediatric ALL patients.

The cyclin-dependent kinase inhibitor 2A (CDKN2A) gene and the cyclin-dependent kinase inhibitor 2B (CDKN2B) gene are two adjacent tumor suppressor genes that are collectively referred to as the CDKN2A/B. The CDKN2A/B is located on the short arm, region 2, band 1 of chromosome 9 (9p21). The CDKN2A encodes two proteins, p16^INK4a^ (p16) and p14^ARF^ (p14), whereas the CDKN2B encodes p15^INK4b^ (p15) [7, 8]. By encoding these three proteins, they play crucial roles in the pathogenesis of leukemia, regulation of the cell cycle, chemosensitivity, and apoptosis. The presence of CDKN2A/B deletion (biallelic or monoallelic) is associated with a lower EFS rate [9]. The CDKN2A often accompanies the CDKN2B with copy number abnormalities, most commonly deletions [10].

Research indicates that the CDKN2A is correlated with the occurrence, development, and prognosis of some tumors, such as ovarian cancer, pancreatic cancer, lung cancer, melanoma, lymphoma, and malignant glioma, and has certain guiding significance for the clinical prognosis and selection of clinical medications for these tumors. In leukemia, studies by Wang et al. [11] demonstrated that deletion of the CDKN2A was associated with poor prognosis in adult ALL patients. Kim et al. [12] found that homozygous deletions of the CDKN2A (p16, p14) and the CDKN2B (p15) was a factor indicating poor prognosis in adult ALL patients, but it did not have a significant impact on prognosis in pediatric ALL patients. Onizuka et al. [13] demonstrated that copy number variations of the CDKN2A/B was a prognostic factor associated with posttransplant relapse in Philadelphia chromosome-positive ALL (ph + ALL). Feng et al. [14] found that deletion of the CDKN2A/B was highly prevalent in pediatric ALL patients and had a detrimental effect on the prognosis of pediatric B-ALL patients, serving as an independent risk factor for poor prognosis.

In summary, the current study [15–18] reveals that there is no fully unified understanding or definitive conclusion regarding the clinical characteristics, biological manifestations, and prognosis of pediatric ALL patients with CDKN2A/B deletion. Therefore, a substantial number of clinical samples and precise experimental data are still needed to analyze the significance of CDKN2A/B deletion in the prognosis of pediatric ALL patients. The aim of this study was to discuss the occurrence and clinical characteristics of CDKN2A/B deletion in pediatric ALL patients, explore whether CDKN2A/B deletion is related to the prognosis of pediatric ALL patients, and provide a new insight for the clinical treatment and prognostic assessment of pediatric ALL patients.

## Materials and methods

Between January 2020 and September 2023, data were collected from 310 pediatric patients diagnosed with ALL at our hospital and treated according to the CCLG-ALL2018 protocol. Among them, 78 patients with CDKN2A/B deletion were ultimately included in the analysis. Additionally, 78 ALL patients without CDKN2A/B deletion, who were diagnosed during the same period, were randomly selected for comparison. The basic data (age, gender, ethnicity), basic clinical information (immunophenotyping, risk stratification, peripheral blood white blood cell count, hemoglobin, platelet count, peripheral blood immature cell proportion), and molecular biological data (chromosome karyotype, combined abnormal genes) at diagnosis were collected. The detailed results of MRD in bone marrow on day 15 and day 33 of induction remission therapy were collected. The prognosis and survival status of both groups of patients were investigated. The cutoff date for follow-up was February 2024, and the total duration ranged from 2 to 48 months. The corresponding statistical methods were used to compare the clinical characteristics, OS and EFS rates between the CDKN2A/B deletion group and the non-deletion group.

### Statistical analysis

SPSS 26.0 and GraphPad Prism 9.0 software were used for statistical analysis. Independent sample *t*-test was used for data that met the normal distribution, and non-parametric rank sum test was used for data that did not meet the normal distribution. Count data were expressed as n (%) and analyzed using Pearson’s chi-square test or Fisher’s exact test when necessary. Measurement data with normal distribution were expressed as mean ± standard deviation, and non-normal distribution were expressed as M (P25, P75). *P* value <0.05 was considered statistically significant. For survival analysis, the Kaplan‒Meier method was used to analyze the overall survival (OS) and event-free survival (EFS) of each group, and the corresponding survival curves were plotted. For prognostic analysis, log-rank univariate regression analysis was first used to screen for factors affecting the prognosis of each group. Factors with *P* < 0.05 were included in the multivariate Cox regression analysis. Ultimately, factors with *P* < 0.05 were considered to have statistically significant differences and were identified as prognostic risk factors.

### Inclusion criteria

(1) Meeting the diagnostic criteria for ALL, patients met the WHO 2016 bone marrow morphology standard, with primitive and immature lymphocytes in the bone marrow accounting for ≥20%, and were diagnosed with ALL based on morphological-immunological-cyto-genetic-molecular (MICM) classification.
(2) Received standardized chemotherapy according to the CCLG-ALL2018 protocol.
(3) Pediatric ALL patients who were identified as CDKN2A/B deletion through high-throughput sequencing for multigene mutation detection in lymphoid tumors.

### Exclusion criteria

(1) Patients who had incomplete MICM classification information at diagnosis or who chose to forgo chemotherapy after diagnosis.
(2) Patients diagnosed with acute mixed lineage leukemia.
(3) Patients who did not receive standardized chemotherapy after diagnosis or were lost to follow-up.
(4) Patients whose complete genetic testing did not detect deletion of the CDKN2A/B.

### Chemotherapy regimen

Chemotherapy was conducted according to the CCLG-ALL2018 (Chinese Children Leukemia Group-ALL2018) treatment protocol. During the chemotherapy process, bone marrow cell morphology and bone marrow minimal residual disease (MRD) were dynamically monitored to assist in assessing the efficacy of chemotherapy and the status of bone marrow remission. Triple intrathecal injections (methotrexate-cytarabine-dexamethasone) were used during the chemotherapy process for the prevention and treatment of central nervous system leukemia (For details, see Table 1).

**TABLE 1:** Simplified overview of the CCLG-B-ALL2018 protocol.

Treatment plan	Low risk (PEG × 4)	Intermediate risk (PEG × 8)	High risk (PEG × 13)
Remission Induction	VDLP (DNR × 2) (PEG-ASP × 2)	VDLP (DNR × 4) (PEG-ASP × 2)	VDLP (DNR × 4) (PEG-ASP × 2)
	1 × CAM	2 × CAML (PEG-ASP × 2)	2 × CAML (PEG-ASP × 2)
Consolidation	Randomized Control 4 × [HD-MTX 2 g/m^2^+6-MP] 4 × [HD-MTX 2 g/m^2^+VD]	Randomized Control 4 × [HD-MTX 5 g/m^2^+6-MP] 4 × [HD-MTX 5 g/m^2^+VD]	2 × (HR-1′, HR-2′, HR- 3′) (PEG-ASP × 6)
Intensification	VDLP (DNR × 3) (PEG-ASP × 2)	VDLP (DNR × 4) (PEG-ASP × 2)	VDLP (DNR × 3) (PEG-ASP × 2)
	1 × CAM	2 × CAML (PEG-ASP × 2)	1 × CAML (PEG-ASP × 1)
Maintenance	6-MP/MTX + VD (4-week Cycle)	6-MP/MTX + VD (4-week Cycle)	6-MP/MTX + VD (4-week Cycle)
Total Treatment Course	Both Males and Females for 2 Years	Females for 2 Years, Males for 2.5 Years	Both Males and Females for 2.5 Years

## Results

### Basic clinical information and immunophenotype at diagnosis

A total of 310 pediatric patients diagnosed with ALL who were treated at our hospital under the CCLG-ALL2018 protocol from January 2020 to September 2023 were included. Among them, 78 patients had CDKN2A/B deletion, accounting for 25.2% (78/310) of the total. Additionally, clinical data from 78 pediatric patients diagnosed during the same period without detected CDKN2A/B deletion were randomly selected for comparison. The details are as follows: (These data are summarized in Table 2).

**TABLE 2:** Basic clinical information and immunophenotype.

Category	Group	CDKN2A/B deletion (N = 78)	Non-deletion of CDKN2A/B (N = 78)	χ2	*P*
Age (year)	—	6.75 (3.23, 11.17)	4.8 (2.92, 6.21)	−2.636	0.008*
Age (year)	<1 year	2 (2.6%)	3 (3.8%)	7.170	0.022*
	1–10 years	51 (65.4%)	64 (82.1%)		
	>10 years	25 (32.1%)	11 (14.1%)		
Sex	Male	50 (64.1%)	41 (52.6%)	2.136	0.144
	Female	28 (35.9%)	37 (47.4%)		
Nationality	Han	45 (57.7%)	52 (66.7%)	1.336	0.248
	Non-Han	33 (42.3%)	26 (33.3%)		
White Blood Cell (×10^9^/L)	—	43.56 (13.51, 131.22)	6.61 (3.58, 25.63)	−5.238	<0.001*
Hemoglobin (g/L)^a^	—	89.55 ± 27.376	80.79 ± 24.491	2.105	0.037*
Platelet (×10^9^/L)	—	57.50 (28.00, 87.00)	43.50 (22.75, 137.75)	−0.399	0.690
Peripheral Blood Immature Cell Proportion (%)	—	61.00 (21.50, 78.25)	30.00 (5.75, 56.50)	−3.999	<0.001*
Immunophenotype	B	47 (60.3%)	74 (94.9%)	26.853	<0.001*
	T	31 (39.7%)	4 (5.1%)		

^a^ Compliance with a normal distribution, represented by mean ± standard deviation.

*: *P* < 0.05, statistically significant difference.

### Sex

CDKN2A/B deletion group: 50 male patients (64.1%, 50/78) and 28 female patients (35.9%, 28/78), with a male-to-female ratio of 1.8:1. CDKN2A/B non-deletion group: 41 male patients (52.6%, 41/78) and 37 female patients (47.4%, 37/78), with a male-to-female ratio of 1.1:1.

### Age

CDKN2A/B deletion group: the median age was 6.75 years (range, 3.23–11.17 years). CDKN2A/B non-deletion group: the median age of the was 4.8 years (range, 2.92–6.21 years).

### Nationality

CDKN2A/B deletion group: 45 Han individuals (57.7%, 45/78) and 33 non-Han individuals (42.3%, 33/78), with a ratio of Han to non-Han individuals of 1.4:1. CDKN2A/B non-deletion group: 52 Han individuals (66.7%, 52/78) and 26 non-Han individuals (33.3%, 26/78), with a ratio of Han to non-Han individuals of 2:1.

### Peripheral blood routine

CDKN2A/B deletion group: the median white blood cell count: 43.56 × 10^9^/L (range, 13.51–131.22) × 10^9^/L; the median platelet count: 57.50 × 10^9^/L (range, 28.00–87.00) × 10^9^/L; the mean hemoglobin level: 89.55 ± 27.376 g/L; the median proportion of peripheral blood immature cells: 61.00% (range, 21.50–78.25%). CDKN2A/B non-deletion group: the median white blood cell count: 6.61 × 10^9^/L (range, 3.58–25.63) × 10^9^/L; the median platelet count: 43.50 × 10^9^/L (range, 22.75–137.75) × 10^9^/L; the mean hemoglobin level: 80.79 ± 24.491 g/L; the median proportion of peripheral blood immature cells: 30.00% (range, 5.75–56.50%).

### Immunophenotype

CDKN2A/B deletion group: 31 T-ALL patients, accounting for 39.7%, and 47 B-ALL patients, accounting for 60.3%. CDKN2A/B non-deletion group: 4 T-ALL patients, accounting for 5.1%, and 74 B-ALL patients, accounting for 94.9%.

Rank sum test was used to compare the differences in age, white blood cell count, hemoglobin levels, platelet count, and the proportion of peripheral blood immature cells between the two groups. The results revealed no statistically significant difference in the platelet count at diagnosis (*P* = 0.690), but statistically significant differences were observed in age *(P* = 0.008), white blood cell count (*P < 0.001)*, hemoglobin level *(P* = 0.037), and the proportion of peripheral blood immature cells (*P < 0.001*). And the results indicated that the age of onset in the CDKN2A/B deletion group was greater than that in the non-deletion group. Both the white blood cell count and the proportion of peripheral blood immature cells at diagnosis were greater in the CDKN2A/B deletion group than in the non-deletion group, suggesting that pediatric patients with CDKN2A/B deletion had a greater tumor burden at diagnosis. The hemoglobin level at diagnosis was slightly greater in the CDKN2A/B deletion group than in the non-deletion group, indicating that the degree of anemia in these patients was milder than that in the non-deletion group.

The Pearson’s chi-squared test was used to compare the basic information of age, sex, and nationality at diagnosis between the two groups. The results revealed no statistically significant differences in sex (*P* = 0.144) or nationality (*P* = 0.248), but there was a statistically significant difference in age (*P* = 0.022). These findings showed that the proportion of patients in the CDKN2A/B deletion group was lower in the 1–10-year-old age group, but was significantly greater in the >10-year-old age group than in the non-deletion group.

The Pearson’s chi-squared test was used to compare the immunophenotype distribution between the two groups, and the results indicated a statistically significant difference (*P* < 0.001). The proportion of T-ALL patients in the CDKN2A/B deletion group was significantly greater than that in the non-deletion group, suggesting that CDKN2A/B deletion occurred more frequently in T-ALL patients than in B-ALL patients.

### Risk stratification and MRD positivity

The CKDN2A/B deletion and the non-deletion groups of pediatric ALL patients were monitored for MRD status and risk stratification during the induction therapy phase on days 15 and 33. MRD positivity was defined as MRD >1 × 10^−1^ on day 15 or MRD >1 × 10^−2^ on day 33. Pearson’s chi-squared test or Fisher’s exact test was used to compare the risk stratification and positive rates of MRD on days 15 and 33 between the two groups. There was no statistically significant difference between the two groups (*P* = 0.409, *P = 0.645, P* = 1.000), suggesting that CDKN2A/B deletion could not be considered to be associated with risk stratification or the positive rate of day 15 MRD or day 33 MRD (These data are summarized in Table 3).

**TABLE 3:** Risk stratification and MRD positivity.

Category	Group	CDKN2A/B deletion (N = 78)	Non-deletion of CDKN2A/B (N = 78)	χ2	*P*
Risk Stratification	Low	1 (1.3%)	4 (5.1%)	1.880	0.409
	Intermediate	55 (70.5%)	55 (70.5%)		
	High	22 (28.2%)	19 (24.4%)		
Day 15 MRD	Positive	12 (15.4%)	10 (12.8%)	0.212	0.645
	Negative	66 (84.6%)	68 (87.2%)		
Day 33 MRD	Positive	1 (1.3%)	2 (2.6%)	△	1.000
	Negative	77 (98.7%)	76 (97.4%)		

△Fisher’s exact test.

### Central nervous system involvement during chemotherapy

Cerebrospinal fluid (CSF) pathological results throughout the chemotherapy process were collected for both groups, with the presence of leukemic cells in the CSF considered central nervous system infiltration (CNS) by leukemia. CDKN2A/B deletion group: 3 patients CNS infiltration (3.8%, 3/78). CDKN2A/B non-deletion group: 2 patients CNS infiltration (2.6%, 2/78). Fisher’s exact test was used for comparison, and the results revealed no statistically significant difference in the incidence of CNS infiltration by leukemia during chemotherapy between the two groups (*P* = 1.000), indicating that there was no association between CDKN2A/B deletion and CNS infiltration.

### Molecular biological characteristics at diagnosis

78 pediatric patients with CDKN2A/B deletion were assessed for bone marrow karyotype and concurrent gene abnormalities at diagnosis. Notably, because patients with chromosomal number abnormalities in the CDKN2A/B deletion group were relatively rare, they were not further classified into subgroups of hypodiploid and hyperdiploid patients for comparison. Instead, they were categorized into three groups based on their bone marrow karyotype: normal chromosomes, numerical abnormalities, and structural abnormalities. Given the vast array of gene detection methods and the increased positive rate of gene abnormalities in the context of next-generation sequencing, numerous genes with unclear prognostic significance for pediatric ALL patients have been identified. Therefore, in this study, concurrent gene abnormalities were classified into four groups based on their prognostic impact: no other gene abnormalities aside from CDKN2A/B deletion, genes associated with a favorable prognosis, genes associated with a poor prognosis, and genes with unclear prognostic impact. For the convenience of the study, several common genes with a clear impact on prognosis were also singled out for individual research.

The Pearson’s chi-squared test was used to compare the prognostic impact of different concurrent gene abnormalities between the two groups. There were no statistically significant differences in the classification of patients with TEL-AML1 (*P* = 0.441), E2A-PBX1 (*P* = 1.000), BCR-ABL1 (*P* = 0.057), or Ph-like-related genes (*P* = 0.615) or SIL-TAL1 gene abnormalities (*P* = 0.477) between the two groups. A statistically significant difference in bone marrow karyotype (*P* = 0.005) was detected between the two groups, and showed that the proportion of chromosomal number abnormalities in the CDKN2A/B deletion group was significantly lower than that in the non-deletion group. A statistically significant difference was also found in the occurrence of BCR-ABL1 positivity combined with IKZF1 deletion (*P* = 0.011) between the two groups, with 8 patients (10.3%) in the deletion group and none in the non-deletion group. These findings suggested that pediatric ALL patients with CDKN2A/B deletion were more likely to exhibit BCR-ABL1 positivity in conjunction with IKZF1 deletion, and all patients were BCR-ABL1 (p190 type) positivity combined with IKZF1 deletion (IK16 type) positive (These data are summarized in Table 4).

**TABLE 4:** Molecular biological characteristics.

Category	Group	CDKN2A/B deletion (N = 78)	Non-deletion of CDKN2A/B (N = 78)	χ2	*P*
Karyotype	Normal Chromosomes	45 (57.7%)	32 (41.0%)	10.605	0.005*
	Numerical Abnormalities	6 (7.7%)	21 (26.9%)		
	Structural Abnormalities	27 (34.6%)	25 (32.1%)		
Concurrent Gene Abnormalities	No Other Gene Abnormalities	10 (12.8%)	14 (17.9%)	4.065	0.254
	With a Favorable Prognosis	7 (9.0%)	13 (16.7%)		
	With a Poor Prognosis	41 (52.6%)	38 (48.7%)		
	With Unclear Prognostic Impact	20 (25.6%)	13 (16.7%)		
Several Common Genes	TEL-AML1	7 (9.0%)	10 (12.8%)	0.594	0.441
	E2A-PBX1	4 (5.1%)	4 (5.1%)	0.000	1.000
	BCR-ABL1	11 (14.1%)	4 (5.1%)	3.614	0.057
	Ph Like	26 (33.3%)	29 (37.2%)	0.253	0.615
	SIL-TAL1	2 (2.6%)	0 (0%)	△	0.477
	BCR-ABL1 Positivity Combined with IKZF1 Deletion	8 (10.3%)	0 (0%)	△	0.011*

△Fisher’s exact test.

*P < 0.05, statistically significant difference.

### Relapse and mortality status

**FIGURE 1:**
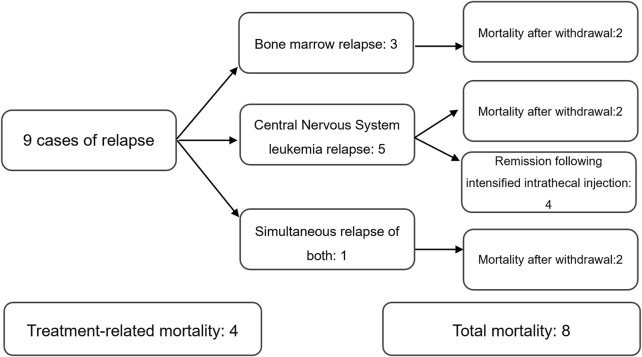
Relapse and mortality status.

Of 78 patients with CDKN2A/B deletion, 9 cases of relapse and 8 cases of mortality. 9 cases of relapse included:3 bone marrow relapses, 5 central nervous system leukemia relapses, and 1 simultaneous relapse of both. 8 cases of mortality included: 4 treatment-related mortalities and 4 mortalities following relapse (Figure 1).

Of 78 patients without CDKN2A/B deletion, 3 cases of relapse, and 2 cases of mortality, both of which were treatment-related mortalities. All 3 cases of relapse were bone marrow relapses.

The Pearson’s chi-squared test was used to compare the relapse and mortality rates between the two groups. The results revealed no statistically significant differences (relapse: *P* = 0.071, mortality: *P* = 0.050), suggesting that CDKN2A/B deletion could not be considered to be related to relapse or mortality in pediatric ALL patients.

### Survival analysis

**FIGURE 2:**
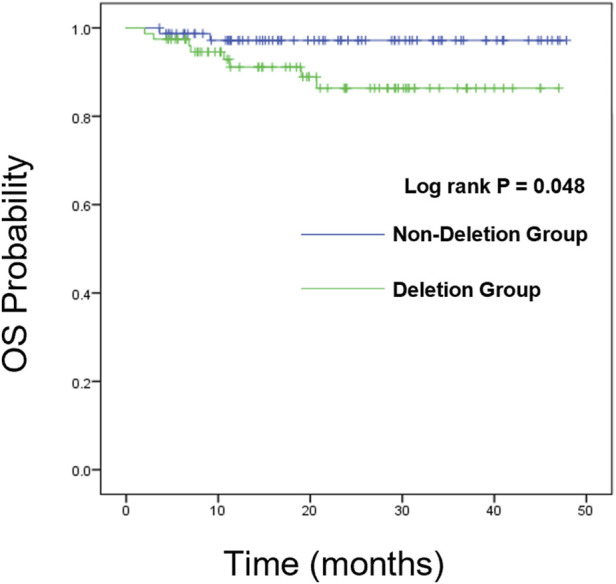
Kaplan-Meier survival curves of OS.

**FIGURE 3:**
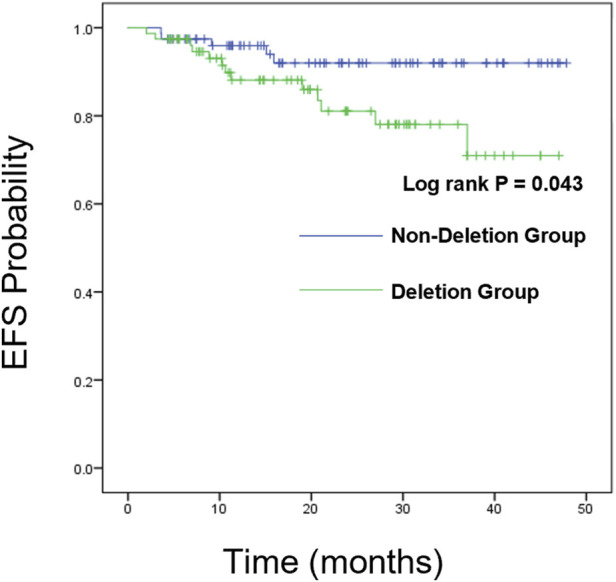
Kaplan-Meier survival curves of EFS.

We further analyzed the overall survival (OS) and event-free survival (EFS) of the two groups. For overall survival analysis, the median time of follow-up in the CDKN2A/B deletion group and non-deletion group was 21 months, 23 months respectively. For event-free survival analysis, the median time of follow-up in the CDKN2A/B deletion group and non-deletion group was 24 months, 23 months respectively. Kaplan-Meier analysis was used to plot the survival curves (see Figures 2, 3).

We compared the OS and EFS rates between the two groups. The Log-rank test showed significant differences in overall OS rate (89.7% vs. 97.4%, *P* = 0.048), 2-year OS rate (89.7% vs. 97.4%, *P* = 0.048), and 3-year OS rate (89.7% vs. 97.4%, *P* = 0.048) between the two groups, indicating that the OS rate of CDKN2A/B deletion group was lower than that of the non-deletion group (These data are summarized in Table 5). Kaplan-Meier analysis was used to plot the survival curves (see Figure 2).

**TABLE 5:** Comparison of OS and EFS rates.

Category	CDKN2A/B deletion (N = 78)	Non-deletion of CDKN2A/B (N = 78)	χ2	*P*
OS rate	70 (89.7%)	76 (97.4%)	3.924	0.048*
1-year OS rate	72 (92.3%)	76 (97.4%)	2.188	0.139
2-year OS rate	70 (89.7%)	76 (97.4%)	3.924	0.048*
3-year OS rate	70 (89.7%)	76 (97.4%)	3.924	0.048*
EFS rate	65 (83.3%)	73 (93.6%)	4.095	0.043*
1-year EFS rate	70 (89.7%)	75 (96.2%)	2.517	0.113
2-year EFS rate	70 (89.7%)	75 (96.2%)	2.517	0.113
3-year EFS rate	66 (84.6%)	73 (93.6%)	3.309	0.069

*P < 0.05, statistically significant difference.

The Log-rank test showed no statistically significant differences between the two groups in 1-year, 2-year, and 3-year EFS rates (*P* > 0.05). However, a statistically significant difference was observed in the total EFS rate (83.3% vs. 93.6%, *P* = 0.043), indicating that the total EFS rate of the CDKN2A/B deletion group was lower than that of the non-deletion group (These data are summarized in Table 5). Kaplan-Meier analysis was used to plot the survival curves (see Figure 3).

### Prognostic factor analysis

#### In the CDKN2A/B deletion group

The statistical results of the OS rates for several factors in the CDKN2A/B deletion group (shown in Table 6) indicated that risk stratification (*P* = 0.027) was a factor affecting the OS rates in pediatric patients with CDKN2A/B deletion. Risk stratification was included in the Cox regression analysis. The results of the Cox regression analysis indicated that risk stratification (95%CI: 1.356∼24.091, *P* = 0.018) was a significant factor affecting the OS rate in pediatric patients with CDKN2A/B deletion.

**TABLE 6:** OS rates for several factors in the CDKN2A/B deletion group.

Category	Group	Total number of cases	Number of events	OS rate	χ2	*P*
Total	—	78	8	89.7%	—	—
Age (year)	<1 year	2	0	100.00%	1.226	0.542
	1–10 years	51	4	92.16%		
	>10 years	25	4	84.00%		
Sex	Male	50	4	92.00%	0.673	0.412
	Female	28	4	85.71%		
Nationality	Han	45	6	86.67%	1.038	0.308
	Non-Han	33	2	93.94%		
Immunophenotype	B	47	5	89.36%	0.047	0.829
	T	31	3	90.32%		
Risk Stratification	Low	1	0	100.00%	7.197	0.027*
	Intermediate	55	3	94.55%		
	High	22	5	77.27%		
Central Nervous System Infiltration	Yes	3	1	66.67%	0.914	0.339
	No	75	7	90.67%		
White Blood Cell ( ×10^9^/L)	<4	7	0	100.00%	2.215	0.330
	4–50	39	6	84.62%		
	>50	32	2	93.75%		
Hemoglobin (g/L)	<30	1	0	100.00%	5.301	0.151
	30–60	7	2	71.43%		
	60–90	35	5	85.71%		
	>90	35	1	97.14%		
Platelet (×10^9^/L)	<50	33	4	87.88%	0.857	0.651
	50–99	27	3	88.89%		
	100–300	18	1	94.44%		
	>300	—	—	—		
Karyotype	Normal	45	5	88.89%	0.583	0.747
	Numerical Abnormalities	6	0	100.00%		
	Structural Abnormalities	27	3	88.89%		
Day 15 MRD	Positive	66	7	89.39%	0.010	0.919
	Negative	12	1	91.67%		
Day 33 MRD	Positive	77	8	89.61%	0.148	0.700
	Negative	1	0	100.00%		
Concurrent Gene Abnormalities	No Other Gene Abnormalities	10	1	90.00%	0.930	0.818
	With a Favorable Prognosis	7	0	100.00%		
	With a Poor Prognosis	41	4	90.24%		
	With Unclear Prognostic Impact	20	3	85.00%		
Concurrent TEL-AML1 Abnormalities	Yes	7	0	100.00%	0.798	0.372
	No	71	8	88.73%		
Concurrent E2A-PBX1 Abnormalities	Yes	4	1	75.00%	0.564	0.453
	No	74	7	90.54%		
Concurrent BCR-ABL1 Abnormalities	Yes	11	2	81.82%	0.776	0.378
	No	67	6	91.04%		
Ph Like	Yes	26	3	88.46%	0.012	0.914
	No	52	5	90.38%		
Concurrent SIT-TALL Abnormalities	Yes	2	1	50.00%	3.271	0.071
	No	76	7	90.79%		
Concurrent BCR-ABL1 Positive Combined with IKZF1 Deletion	Yes	8	1	87.50%	0.054	0.816
	No	70	7	90.00%		

*P < 0.05, statistically significant difference.

The statistical results of EFS rates for several factors in the CDKN2A/B deletion group (shown in Table 7) indicated that risk stratification was a factor affecting the EFS rates in pediatric patients with CDKN2A/B gene deletion (*P* = 0.010). Risk stratification was included in the Cox regression analysis. The results of the Cox regression analysis indicated that risk stratification (95%CI: 1.567∼14.290, *P* = 0.006) was a significant factor affecting the EFS rate in pediatric patients with CDKN2A/B gene deletion.

**TABLE 7:** EFS rates for several factors in the CDKN2A/B deletion group.

Category	Group	Total number of cases	Number of events	EFS rate	χ2	*P*
Total	—	78	13	83.3%	—	—
Age (year)	<1 year	2	1	50.00%	1.190	0.552
	1–10 years	51	7	86.27%		
	>10 years	25	5	80.00%		
Sex	Male	50	8	84.00%	0.009	0.923
	Female	28	5	82.14%		
Nationality	Han	45	9	80.00%	0.918	0.338
	Non-Han	33	4	87.88%		
Immunophenotype	B	47	6	87.23%	1.235	0.267
	T	31	7	77.42%		
Risk Stratification	Low	1	0	100.00%	9.164	0.010
	Intermediate	55	6	89.09%		
	High	22	7	68.18%		
Central Nervous System Infiltration	Yes	3	2	33.33%	3.062	0.080
	No	75	11	85.33%		
White Blood Cell (×10^9^/L)	<4	7	1	85.71%	0.129	0.937
	4–50	39	7	82.05%		
	>50	32	5	84.38%		
Hemoglobin (g/L)	<30	1	0	100.00%	5.705	0.127
	30–60	7	3	57.14%		
	60–90	35	7	80.00%		
	>90	35	3	91.43%		
Platelet (×10^9^/L)	<50	33	8	75.76%	3.238	0.198
	50–99	27	4	85.19%		
	100–300	18	1	94.44%		
	>300	—	—	—		
Karyotype	Normal	45	9	80.00%	2.654	0.265
	Numerical Abnormalities	6	0	100.00%		
	Structural Abnormalities	27	4	85.19%		
Day 15 MRD	Positive	66	12	81.82%	0.325	0.569
	Negative	12	1	91.67%		
Day 33 MRD	Positive	77	13	83.12%	0.350	0.554
	Negative	1	0	100.00%		
Concurrent Gene Abnormalities	No Other Gene Abnormalities	10	1	90.00%	2.027	0.567
	With a Favorable Prognosis	7	0	100.00%		
	With a Poor Prognosis	41	7	82.93%		
	With Unclear Prognostic Impact	20	5	75.00%		
Concurrent TEL-AML1 Abnormalities	Yes	7	0	100.00%	1.507	0.220
	No	71	13	81.69%		
Concurrent E2A-PBX1 Abnormalities	Yes	4	1	75.00%	0.112	0.737
	No	74	12	83.78%		
Concurrent BCR-ABL1 Abnormalities	Yes	11	2	81.82%	0.000	0.986
	No	67	11	83.58%		
Ph Like	Yes	26	3	88.46%	1.213	0.271
	No	52	10	80.77%		
Concurrent SIT-TALL Abnormalities	Yes	2	1	50.00%	1.674	0.196
	No	76	12	84.21%		
Concurrent BCR-ABL1 Positive Combined with IKZF1 Deletion	Yes	8	1	87.50%	0.105	0.746
	No	70	12	82.86%		

*P < 0.05, statistically significant difference.

These findings suggested that the greater the risk stratification of pediatric patients with CDKN2A/B gene deletion, the greater the possibility of relapse or death.

#### In the CDKN2A/B non-deletion group

The statistical results of OS rates for several factors in the CDKN2A/B non-deletion Group (shown in Table 8) indicated that the immunophenotype (*P* = 0.001) and the positivity rate of MRD on day 33 (*P* < 0.001) were factors affecting the OS rates in pediatric patients without CDKN2A/B gene deletion. The immunophenotype and positivity rate of MRD on day 33 were included in the multivariate Cox regression analysis. The results of the Cox regression analysis indicated that neither the immunophenotype (*P* = 0.977) nor the positivity rate of MRD on day 33 (*P* = 0.965) were significant factors affecting the OS rates in pediatric patients without CDKN2A/B gene deletion.

**TABLE 8:** OS rates for several factors in the CDKN2A/B non-deletion group.

Category	Group	Total number of cases	Number of events	OS rate	χ2	*P*
Total	—	78	2	97.4%	—	—
Age (year)	<1 year	3	0	100.00%	2.334	0.311
	1–10 years	64	1	98.44%		
	>10 years	11	1	90.91%		
Sex	Male	41	0	100.00%	2.319	0.128
	Female	37	2	94.59%		
Nationality	Han	52	2	96.15%	1.057	0.304
	Non-Han	26	0	100.00%		
Immunophenotype	B	74	1	98.65%	10.274	0.001*
	T	4	1	75.00%		
Risk Stratification	Low	4	0	100.00%	5.645	0.059
	Intermediate	55	0	100.00%		
	High	19	2	89.47%		
Central Nervous System Infiltration	Yes	2	0	100.00%	0.042	0.837
	No	76	2	97.37%		
White Blood Cell ( ×10^9^/L)	<4	9	1	88.89%	4.346	0.114
	4–50	18	0	100.00%		
	>50	51	1	98.04%		
Hemoglobin (g/L)	<30	1	0	100.00%	0.623	0.891
	30–60	14	0	100.00%		
	60–90	38	1	97.37%		
	>90	25	1	96.00%		
Platelet (×10^9^/L)	<50	60	1	98.33%	6.701	0.082
	50–99	6	1	83.33%		
	100–300	10	0	100.00%		
	>300	2	0	100.00%		
Karyotype	Normal	32	0	100.00%	3.773	0.152
	Numerical Abnormalities	21	0	100.00%		
	Structural Abnormalities	25	2	92.00%		
Day 15 MRD	Positive	68	1	98.53%	2.110	0.146
	Negative	10	1	90.00%		
Day 33 MRD	Positive	76	1	98.68%	16.141	<0.001*
	Negative	2	1	50.00%		
Concurrent Gene Abnormalities	No Other Gene Abnormalities	14	0	100.00%	1.996	0.573
	With a Favorable Prognosis	13	0	100.00%		
	With a Poor Prognosis	38	2	94.74%		
	With Unclear Prognostic Impact	13	0	100.00%		
Concurrent TEL-AML1 Abnormalities	Yes	10	0	100.00%	0.310	0.578
	No	68	2	97.06%		
Concurrent E2A-PBX1 Abnormalities	Yes	4	0	100.00%	0.103	0.748
	No	74	2	97.30%		
Concurrent BCR-ABL1 Abnormalities	Yes	4	0	100.00%	0.103	0.748
	No	74	2	97.30%		
Ph Like	Yes	29	2	93.10%	3.255	0.071
	No	49	0	100.00%		
Concurrent SIT-TALL Abnormalities	No	78	2	97.44%	—	—
Concurrent BCR-ABL1 Positive Combined with IKZF1 Deletion	No	78	2	97.44%	—	—

*: *P* < 0.05, statistically significant difference.

The statistical results related to EFS rates for several factors in the CDKN2A/B non-deletion Group (shown in Table 9) indicated that the immunophenotype (*P* < 0.001) and the positivity rate of MRD on day 33 (*P* = 0.012) were factors affecting the EFS rates in pediatric patients without CDKN2A/B gene deletion. The immunophenotype and positivity rate of MRD on day 33 were included in the multivariate Cox regression analysis. The results of the Cox regression analysis indicated that the T-cell immunophenotype (95%CI: 1.728∼162.834, *P* = 0.015) was a significant factor affecting the EFS rate in pediatric patients without CDKN2A/B deletion. These findings suggested that pediatric patients without CDKN2A/B deletion had a greater possibility of relapse or death when they were diagnosed with T-ALL.

**TABLE 9:** EFS rates for several factors in the CDKN2A/B non-deletion group.

Category	Group	Total number of cases	Number of events	EFS rate	χ2	*P*
Total	—	78	5	93.6%	—	—
Age (year)	<1 year	3	0	100.00%	3.267	0.195
	1–10 years	64	3	95.31%		
	>10 years	11	2	81.82%		
Sex	Male	41	3	92.68%	0.104	0.747
	Female	37	2	94.59%		
Nationality	Han	52	4	92.31%	0.525	0.469
	Non-Han	26	1	96.15%		
Immunophenotype	B	74	3	95.95%	15.091	<0.001*
	T	4	2	50.00%		
Risk Stratification	Low	4	0	100.00%	3.030	0.220
	Intermediate	55	2	96.36%		
	High	19	3	84.21%		
Central Nervous System Infiltration	Yes	2	0	100.00%	0.111	0.739
	No	76	5	93.42%		
White Blood Cell (×10^9^/L)	<4	9	1	88.89%	2.557	0.278
	4–50	18	0	100.00%		
	>50	51	4	92.16%		
Hemoglobin (g/L)	<30	1	0	100.00%	1.528	0.676
	30–60	14	0	100.00%		
	60–90	38	3	92.11%		
	>90	25	2	92.00%		
Platelet (×10^9^/L)	<50	60	4	93.33%	2.279	0.517
	50–99	6	1	83.33%		
	100–300	10	0	100.00%		
	>300	2	0	100.00%		
Karyotype	Normal	32	1	96.88%	1.191	0.551
	Numerical Abnormalities	21	1	95.24%		
	Structural Abnormalities	25	3	88.00%		
Day 15 MRD	Positive	68	4	94.12%	0.266	0.606
	Negative	10	1	90.00%		
Day 33 MRD	Positive	76	4	94.74%	6.345	0.012*
	Negative	2	1	50.00%		
Concurrent Gene Abnormalities	No Other Gene Abnormalities	14	0	100.00%	2.411	0.492
	With a Favorable Prognosis	13	0	100.00%		
	With a Poor Prognosis	38	4	89.47%		
	With Unclear Prognostic Impact	13	1	92.31%		
Concurrent TEL-AML1 Abnormalities	Yes	10	0	100.00%	0.857	0.355
	No	68	5	92.65%		
Concurrent E2A-PBX1 Abnormalities	Yes	4	0	100.00%	0.267	0.605
	No	74	5	93.24%		
Concurrent BCR-ABL1 Abnormalities	Yes	4	0	100.00%	0.157	0.692
	No	74	5	93.24%		
Ph Like	Yes	29	3	89.66%	0.937	0.333
	No	49	2	95.92%		
Concurrent SIT-TALL Abnormalities	No	78	5	93.60%	—	—
Concurrent BCR-ABL1 Positive Combined with IKZF1 Deletion	No	78	5	93.60%	—	—

*P < 0.05, statistically significant difference.

## Discussion

### CDKN2A/B deletion rate and clinical characteristics

In this study, the male-to-female ratio among pediatric ALL patients was 1.8:1, slightly higher than the overall male-to-female ratio of 1.5:1 reported in epidemiological studies of ALL in China. Overseas studies indicated that the rate of CDKN2A/B deletion in pediatric ALL patients is between 15% and 35% [16]. Several studies have also reported a 44% overall frequency of CDKN2A/B deletion in pediatric ALL patients [19]. In the study by Steeghs et al., the rate of CDKN2A/B deletion was 33% [20]. In this study of 310 pediatric ALL patients, 78 patients with CDKN2A/B deletion were detected, accounting for 25.2%, slightly lower than the rates reported in the studies mentioned above. This discrepancy may be related to the smaller sample size or racial differences between countries, and further research with an expanded sample size is needed for confirmation. In this study, statistical analysis revealed a significant difference in the occurrence of CDKN2A/B deletion between the two groups in terms of immunophenotype (*P* < 0.001), with a greater proportion of T-ALL patients in the CDKN2A/B deletion group than in the non-deletion group. The proportion of patients with CDKN2A/B deletion in T-ALL patients was greater than that in B-ALL patients. This was consistent with the findings of studies by Agarwal et al. [21] and Sulong [22]. In terms of age at diagnosis, the proportion of CDKN2A/B deletion group was higher in the age >10 years than that in the non-deletion group (*P* = 0.022), which was consistent with the findings of Agarwal et al. [21] that deletion was more likely to occur in older children. Additionally, the white blood cell count and the proportion of peripheral blood immature cells at diagnosis were greater in the deletion group than in the non-deletion group. This finding was similar to the results of Kathiravan et al. [19], suggesting that pediatric patients with CDKN2A/B deletion had a greater tumor burden at diagnosis. In summary, this study revealed that pediatric patients with CDKN2A/B deletion generally present with the following clinical characteristics: a greater proportion of T-ALL patients, a higher median white blood cell count at diagnosis, and a greater proportion in the age >10 years. T-ALL, a white blood cell count of ≥50 × 10^9^/L, age ≥1 year was classified as intermediate risk according to the risk stratification of the CCLG-ALL2018 protocol. In this study, there was a statistically significant difference in bone marrow karyotype between the two groups, indicating that the proportion of chromosomal number abnormalities in the CDKN2A/B deletion group was significantly lower than that in the non-deletion group. This finding was not consistent with the study by González-Gil [16], which reported a relatively low frequency of CDKN2A/B deletion in patients with a hyperdiploid karyotype, which was associated with a favorable prognosis. Furthermore, statistical analysis revealed that the CDKN2A/B deletion group was prone to BCR-ABL1 positivity concurrent with IKZF1 deletion, and all the patients were BCR-ABL1 (p190 type) positivity concurrently with IKZF1 deletion (IK16 type) positive. No cases of BCR-ABL1 positivity concurrent with IKZF1 deletion were found in the non-deletion group. This finding was not entirely consistent with the study by González-Gil [16], which suggested that CDKN2A/B deletion was more frequently observed in high-risk pediatric patients, especially those with BCR-ABL1 positivity. However, both BCR-ABL1 positivity and IKZF1 deletion are markers of poor prognosis. A larger sample size is needed to further investigate whether CDKN2A/B deletion is associated with BCR-ABL1 positivity combined with IKZF1 deletion and how the coexistence of the three affects the prognosis of pediatric ALL patients. Research by Williams et al. [23] indicated that in the treatment of BCR-ABL1 positive B-ALL with imatinib, the presence of ARF deletion at the p14 locus encoded by the CDKN2A can affect sensitivity to imatinib, leading to drug resistance and poorer treatment outcomes. However, the mechanism remains unclear. The use of imatinib in combination with a JAK kinase inhibitor may be beneficial for the treatment of B-ALL patients with BCR-ABL1 positivity combined with ARF locus deletion. Iacobucci et al. [24] have shown that the presence of IKZF1 deletion combined with deletion of the ARF locus encoded by the CDKN2A was associated with poorer prognosis and higher risk of relapse and can lead to resistance to TKIs targeted therapy in adults with BCR-ABL1 positive B-ALL. In pediatric ALL, more samples and relevant experimental data are needed to explore whether deletion of the CDKN2A/B or the encoded locus is related to resistance to TKIs in BCR-ABL1-positive patients, and may be able to guide the selection of targeted therapies for BCR-ABL1 positive pediatric ALL patients.

### Survival analysis and prognostic factor analysis

Under the CCLG-ALL2008 protocol in China, the 5-year OS and EFS rates for pediatric ALL patients were reported to be 85.3% and 79.9%, respectively [25]. In this study, the overall OS and EFS rates were 89.7%, and 83.3%, respectively. The 1-year OS and EFS rates were 92.3% and 89.7%, respectively. The 2-year OS and EFS rates were 89.7% and 89.7%, respectively. The 3-year OS and EFS rates were 89.7% and 84.6%, respectively. These rates were lower than those of the non-deletion group (For details see Table 5). Statistical analysis revealed significant differences in the overall OS rates, 2-year OS rates, 3-year OS rates, and overall EFS rates between the two groups of pediatric ALL patients (*P* < 0.05), indicating that CDKN2A/B deletion was associated with lower OS and EFS rates. These findings were generally consistent with the results of studies by Agarwal [21] and Kathiravan [19], which revealed that pediatric ALL patients with CDKN2A/B deletion had a greater risk and lower event-free survival rate. Owing to the relatively short follow-up period in this study, further analyses with more samples and extended follow-up time are needed. Statistical analysis of prognostic factors revealed that risk stratification was a significant factor affecting the OS and EFS rates in pediatric patients with CDKN2A/B deletion (*P* = 0.018, *P* = 0.06). For patients with CDKN2A/B deletion, the higher the risk stratification level was, the greater the possibility of relapse or death.

Currently, there is still variability in the prognosis of pediatric ALL patients related to the CDKN2A/B, which may be due to the deletion of the CDKN2A/B being associated with specific genetics, with the frequency of CDKN2A/B deletion varying significantly depending on combination of other genes [22]. In this study, CDKN2A/B deletion combined with a number of risk stratification factors for intermediate risk, and it was easy to combine BCR-ABL1 positivity and IKZF1 deletion, but there was no statistical significance in risk stratification, which may be related to the small sample size of this study. With the progress of gene research in the field of leukemia and the further expansion of sample size, CDKN2A/B deletion may be included as a criterion for intermediate risk in the stratification of pediatric ALL patients.

## Conclusion

At diagnosis, pediatric patients with CDKN2A/B deletion had higher peripheral blood white blood cell counts and proportions of immature cells in the peripheral blood. The age at diagnosis was older in the deletion group, with a greater proportion in the age group over 10 years old.

Compared with that in pediatric patients with B-ALL, deletion of the CDKN2A/B occurred more frequently in pediatric patients with T-ALL.

CDKN2A/B deletion was more likely to result in BCR-ABL1 positivity combined with IKZF1 deletion.

Pediatric patients with CDKN2A/B deletion had lower OS and EFS rates, indicating that CDKN2A/B deletion was one of the factors influencing poor prognosis.

## Data Availability

The original contributions presented in the study are included in the article, further inquiries can be directed to the corresponding authors.
